# Heteroepitaxial Growth of *β*‐Ga_2_O_3_ on Diamond (111) via Radio Frequency Magnetron Sputtering: Mechanistic Insights from Scanning/Transmission Electron Microscopy

**DOI:** 10.1002/smll.202507322

**Published:** 2025-10-16

**Authors:** Itsuki Misono, Sho Nekita, Hongye Gao, Sreenath Mylo Valappil, Yixin Wang, Yuto Ikegami, Yuki Katamune, Hiroshi Naragino, Phongsaphak Sittimart, Shinya Ohmagari, Abdelrahman Zkria, Satoshi Hata, Tsuyoshi Yoshitake

**Affiliations:** ^1^ Interdisciplinary Graduate School of Engineering Sciences Kyushu University 6‐1 Kasuga‐koen Kasuga Fukuoka 816–8580 Japan; ^2^ The Ultramicroscopy Research Center Kyushu University 744 Motooka, Nishi‐ku Fukuoka Fukuoka 819‐0395 Japan; ^3^ Department of Electrical and Electronic Engineering Kyushu Institute of Technology 1‐1 Sensuicho, Tobata Kitakyushu Fukuoka 804–8550 Japan; ^4^ Department of Electrical and Computer Engineering The Sirindhorn International Thai‐German Graduate School of Engineering (TGGS) King Mongkut's University of Technology North Bangkok Bangkok 10800 Thailand; ^5^ Sensing System Research Center National Institute of Advanced Industrial Science and Technology (AIST) 807‐1 Shuku‐machi Tosu Saga 841‐0052 Japan; ^6^ Department of Physics Faculty of Science Aswan University Aswan 81528 Egypt

**Keywords:** domain structure, Ga_2_O_3_/diamond, heteroepitaxy, transmission electron microscopy

## Abstract

This study delves into the comprehensive microstructural analysis of heteroepitaxial *β*‐Ga_2_O_3_ thin films grown on single‐crystal diamond (111) wafers by radio frequency magnetron sputtering. The heterostructure is probed through a scanning/transmission electron microscope and succeeded in direct observation of *β*‐Ga_2_O_3_ <010>||diamond [1$\bar{1}$0] and *β*‐Ga_2_O_3_ <132>||diamond [1$\bar{1}$0] heteroepitaxial interfaces, which up to now have only has been inferred through indirect analyzing methods based on X‐ray diffraction. Fast Fourier Transformation (FFT) patterns of corresponding oriented interfaces exhibited virtual overlapping for the substrate and the epilayer, authenticating the robust epitaxial arrangement. Furthermore, this study elucidates the discrete growth modes of *β*‐Ga_2_O_3_ <010> and <132> domains arising from the asymmetric hexagonal C─O lattice matching, through 4D‐STEM analysis and subsequent virtual dark‐field image reconstruction. Strategies for the improvement of crystallinity, surface morphology, and facile thickness controllability of the β‐Ga_2_O_3_ epilayer through optimizing the RF power, facilitating a low re‐evaporation rate of the film, are discussed. This finding underscores the importance of domain control in the improved epitaxial quality of *β*‐Ga_2_O_3_ that can inform the development of budget‐friendly and scalable *β*‐Ga_2_O_3_/diamond heterostructures for advanced functional devices.

## Graphical abstract

This study achieved the first direct observation of β‐Ga_2_O_3_/diamond (111) interfaces grown via radio frequency magnetron sputtering (RFMS). Distinct epitaxial orientations showed differing growth modes: <010> domains grew columnarly, while <132> domains grew laterally. Controlling these domains is crucial for high‐quality thin films. The findings support RFMS as a cost‐effective method for fabricating β‐Ga_2_O_3_/diamond structures for thermal and power applications.

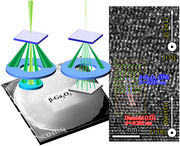

## 1 Introduction

In recent years, a significant body of research has been dedicated to investigating the prospective utilization of *β*‐Ga_2_O_3_ in next‐generation power electronics and sensing devices.^[^ ^1^, ^2^, ^3^, ^4^ ^]^ The material's noteworthy physical properties include a wide bandgap of 4.5–4.9 eV, a high breakdown electric field (≈8 MV cm^−1^), profound chemical stability, and radiation resistance.^[^ ^5^, ^6^, ^7^ ^]^ Furthermore, the reduced manufacturing cost of *β*‐Ga_2_O_3_ single‐crystal wafers renders it a more cost‐effective alternative to commercially viable power device materials, such as SiC and GaN.^[^ ^8^ ^]^ Notwithstanding the potential material properties of *β*‐Ga_2_O_3_, which are excellent, its practical deployment is constrained by two factors. First, the material's low thermal conductivity (10–30 W m^−1^ K^−1^) poses a significant challenge. Second, the material's inability to achieve p‐type electrical conductivity due to hole self‐trapping limits its applications to unipolar devices.^[^ ^9^, ^10^ ^]^

In the context of advanced devices based on *β*‐Ga_2_O_3_, crystalline diamonds are regarded as the optimal complementary substrate material to address the aforementioned limitations.^[^ ^11^, ^12^, ^13^ ^]^ This is attributed to their high thermal conductivity (≈2000 W m^−1^ K^−1^), wide band gap (5.47 eV), and effective p‐type doping strategy.^[^ ^10^, ^14^, ^15^ ^]^ Recent research has demonstrated the compelling potential of integrating *β*‐Ga_2_O_3_ with diamond layers, improving the device functionality.^[^ ^16^, ^17^ ^]^ An ideal Ga_2_O_3_/diamond integration offers a multifaceted approach to enhancing the performance of advanced heterojunctions and high‐temperature deployable devices through quality thermal transport and reduced interface trap state density.

To this end, in 2020, Matsumae et al. have demonstrated atomically bonded void‐free *β*‐Ga_2_O_3_ (010)/diamond (111) interfaces through thermal‐assisted direct bonding of surface functionalized individual components.^[^ ^18^ ^]^ Despite the absence of direct heteroepitaxial growth, the low‐temperature direct bonding technique has demonstrated efficacy in the formation of diamond (100)*/β*‐Ga_2_O_3_ (100) p‐n junctions, exhibiting enhanced junction parameters in comparison to those achieved through van der Waals integration.^[^ ^6^, ^19^ ^]^ In a similar vein, Cheng et al. (2023) have indicated the significance of Ga_2_O_3_ /diamond integration for active thermal management in future devices. Their findings suggest that covalently bonded polycrystalline Ga_2_O_3_/diamond interfaces by atomic layer deposition (ALD) exhibit nearly ten‐fold higher thermal boundary conductance (TBC) compared to van der Waals interfacial bonding.^[^ ^20^ ^]^ Evolving toward the heteroepitaxial integration of the two materials, Nandi et al. in 2023 exhibited a two‐step growth process of ($\bar{2}$01) oriented *β*‐Ga_2_O_3_ by metal organic chemical vapor deposition (MOCVD) on diamond (001) substrates, comprising two sets of epitaxial grain variants, which hampers the formation of a single crystalline film.^[^ ^21^ ^]^ Similarly, Xu et al. also demonstrated a robust out‐of‐plane epitaxial relationship between *β*‐Ga_2_O_3_ and diamond (001) integrated using pulsed laser deposition (PLD).^[^ ^22^ ^]^ Additionally, device strategies that incorporate *β*‐Ga_2_O_3_/diamond heterostructures are undergoing active development to facilitate practical applications in domains such as power electronics and radiation detection.^[^ ^23^, ^24^, ^25^ ^]^

Toward acknowledging the scenario of robust interfacial integration, we have successfully demonstrated the heteroepitaxial growth of undoped *β*‐Ga_2_O_3_ thin films on diamond (111) substrates by RF magnetron sputtering (RFMS) in our recent research.^[^ ^26^ ^]^ However, despite the successful demonstration of the epitaxial growth of *β*‐Ga_2_O_3_ thin films on diamond (111) through indirect analysis methods, an exhaustive understanding of the growth mechanism and an explicit formation of the heteroepitaxial interface need to be clarified and revealed. Furthermore, the preceding study suggested that enhancing the growth efficiency of *β*‐Ga_2_O_3_ thin films on a diamond substrate using RFMS, including parameters such as growth rate and film morphology, is a promising avenue for innovative research.

In this study, the RFMS‐grown *β*‐Ga_2_O_3_/diamond (111) heterostructure is comprehensively evaluated using scanning/transmission electron microscopy (S/TEM). Formation of the heteroepitaxial *β*‐Ga_2_O_3_/diamond (111) interface is clarified. To the best of our available information, this investigation represents the world‐first instance of periodic atomic arrangement at the *β*‐Ga_2_O_3_/diamond (111) heteroepitaxial interface fabricated using RFMS. In addition, a notable improvement in the efficiency of *β*‐Ga_2_O_3_ thin film deposition on diamond (111) has been attained by adjusting the RF power. This reduction has led to the suppression of desorption of adatoms and secondary sputtering, consequently enhancing the deposition rate and film morphology.

## 2 Results and Discussion

**Figure 1:**
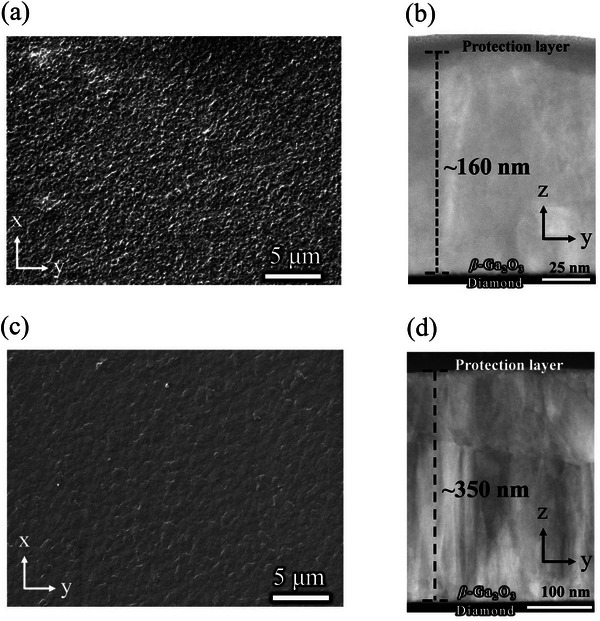
The high magnification SEM image of the *β*‐Ga_2_O_3_ film surface fabricated at a) 50 W and c) 35 W. The cross‐sectional ADF‐STEM image of *β*‐Ga_2_O_3_ film fabricated at b) 50 W and d) 35 W.

**Figure** **1** shows microscopic images of the surface and cross‐sectional morphologies of the *β*‐Ga_2_O_3_ film fabricated at RF powers of 50 and 35 W. Interestingly, the thickness of the *β*‐Ga_2_O_3_ film nearly doubled at 35 W with a relatively shorter deposition time. In most cases, it has been reported that decreasing the RF power results in a low deposition rate, consequently decreasing the thickness of the film.^[^ ^27^, ^28^, ^29^ ^]^ However, in cases where the growth power is higher for the re‐evaporation rate to overcome the growth rate, the film thickness decreases.^[^ ^30^, ^31^ ^]^ Thus, it can be inferred that decreasing the growth power to 35 W resulted in lower re‐evaporation rates of adatoms by reducing the kinetic energy of the sputtered species below the binding force threshold within the *β*‐Ga_2_O_3_ film.^[^ ^32^ ^]^

Additionally, our recent report indicated an inverse relationship between the thickness of the heteroepitaxial *β*‐Ga_2_O_3_ film and the substrate temperature. In light of this scenario, RF power‐associated thermal changes of sputtered species can also contribute to the re‐evaporation of the adatoms. In addition, the surface structure demonstrated substantial alterations, wherein the development of mountain‐like crystals is impeded at 35 W. Consequently, it can be deduced that decreasing the RF power promotes a layer‐by‐layer growth pattern, contrasting with the Stranski–Krastanov (S–K) growth mode for 50 W grown epilayer as mentioned in our first report.^[^ ^26^ ^]^ The film deposition rates were calculated to be 2.2 and 7.3 nm h^−1^ for 50 and 35 W, respectively. In scenarios of device fabrication, the facile controllability of film thickness and smooth film morphology play crucial roles. Despite reducing the RF power to 35 W, which has an improving effect on the aforementioned factors, it is imperative to acknowledge its effects on film orientation, chemical composition, and crystallinity.

**Figure 2:**
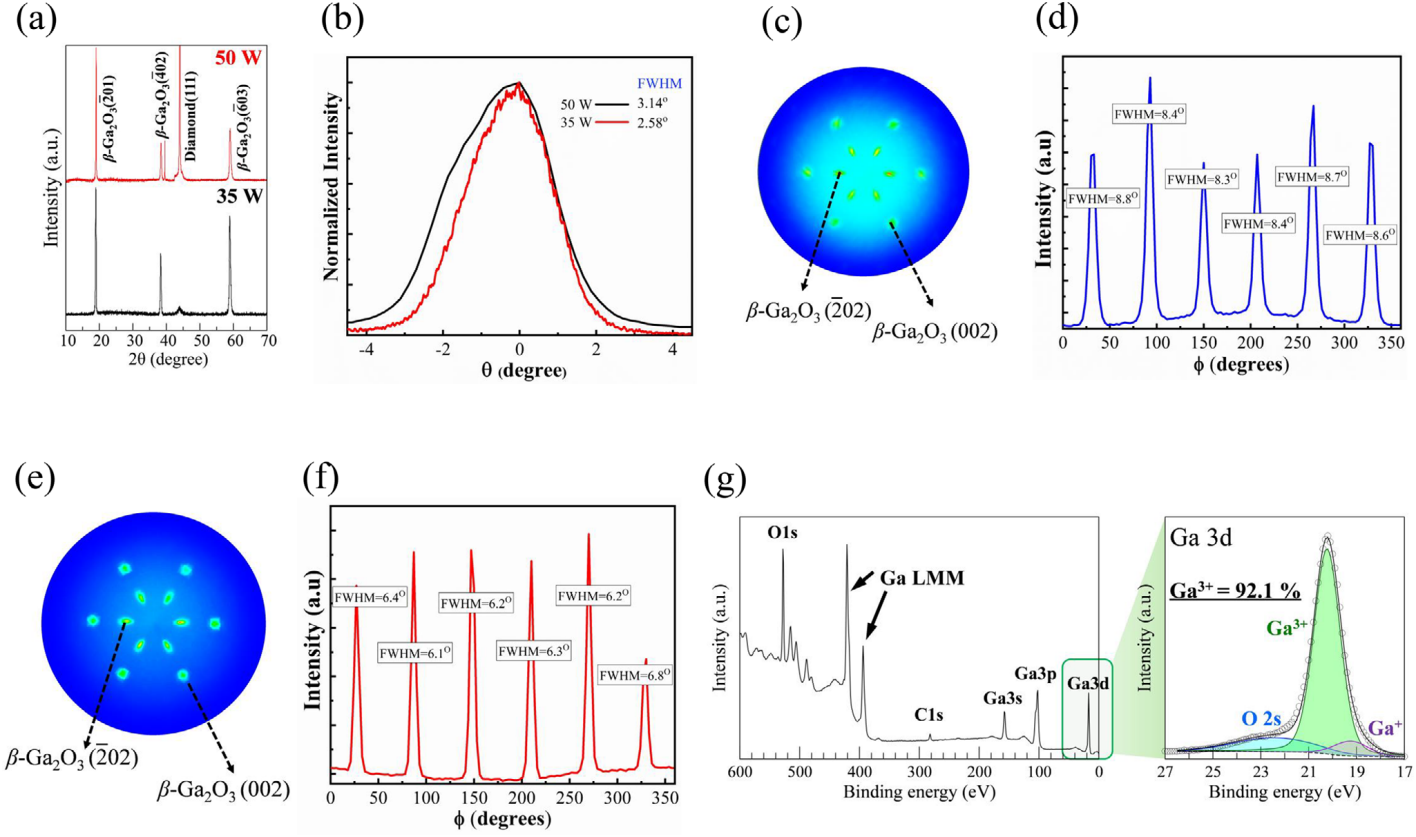
a) θ‐2θ XRD scans of *β*‐Ga_2_O_3_/diamond (111) heterostructures fabricated at 50 and 35 W. b) XRD rocking curves of β‐Ga2O3 (2̅01) peak of respective samples. X‐ray pole figure stereographic projections of β‐Ga_2_O_3_/diamond (111) heterostructures fabricated at c) 50 W e) 35 W, and their corresponding rotational intensity mapping d,f) at ψ = 50°. g) XPS wide spectrum and Ga 3d narrow spectrum of a 35 W grown *β*‐Ga_2_O_3_ film.

**Figure** **2** shows the comparative crystallinity assessments of the 50 and 35 W grown films through X‐ray diffraction methods. As clearly seen in the patterns, only *β*‐Ga_2_O_3_ {$\bar{2}$01} family diffraction peaks, together with the diamond (111) diffraction peak, were observed for both specimens. These {$\bar{2}$01} family peaks consist of ($\bar{2}$01), ($\bar{4}$02) and ($\bar{6}$03), appearing at 2*θ* of 18.94°, 38.44°, and 59.18°, respectively. The {$\bar{2}$01} family planes imply that Ga_2_O_3_ thin films with monoclinic phase were deposited on the diamond (111) substrates.^[^ ^17^, ^25^ ^]^

However, *θ*‐2*θ* analysis only informs about the out‐of‐plane stacking. Therefore, pole‐figure stereographic projection was carried out to confirm the intact in‐plane orientation. Figure 2c,e are pole‐figure stereographic projections of *β*‐Ga_2_O_3_ deposited at RF powers of 50 and 35 W, respectively. In both cases, six‐fold symmetry spots are observed, which are split by *ϕ* ≈ 60° corresponding to *β*‐Ga_2_O_3_ {$\bar{2}$02} at ψ = 22.2° ± 2° and *β*‐Ga_2_O_3_ {002} at ψ = 50° ± 2. These results indicate that *β*‐Ga_2_O_3_ thin films were epitaxially deposited on diamond (111) substrates with intact in‐plane and out‐of‐plane orientations.^[^ ^33^ ^]^ Additionally, the pole figure domain spots of the 35 W grown sample become slightly narrower with an improved signal‐to‐noise ratio than the 50 W‐grown sample. This can be evident from the narrowing trend of FWHM of the rotational intensity distribution of the pole figure spots at ψ = 50°, as shown in Figure 2d,f. Also, the reduced signal‐to‐noise ratio of the pole figure spots can be understood by comparing the baselines of the intensity distribution. This result suggests that lower RF power improved the crystallinity of the *β*‐Ga_2_O_3_ deposited by RFMS. Confirming this observation, the FWHM of *β*‐Ga_2_O_3_ ($\bar{2}$01) rocking curve decreased to 82% when the RF power changed from 50 to 35 W, as shown in Figure 2b.

Figure 2g shows the XPS scans indicating the chemical bonding state of the *β*‐Ga_2_O_3_ film deposited at RF power of 35 W. The chemical composition of the film did not exhibit notable differences in elemental peaks. Narrow scan of Ga 3d region indicates the existence of Ga^+^ component peaks related to oxygen vacancies, which are the typical point defects in *β*‐Ga_2_O_3_ film.^[^ ^34^ ^]^ However, the deconvoluted area ratio of Ga^3+^ / (Ga3^+^ + Ga^+^) has increased from 89.7% to 92.1%, suggesting a lower RF may benefit the suppression of oxygen vacancies by hindering excessive ion bombardment during the film growth. At this stage, there may be an effect from the different thicknesses of the *β*‐Ga_2_O_3_ epilayers in the comparative crystallinity analysis using X‐ray diffraction, as the X‐ray path length through the material volume affects the diffraction signal yield. Toward more microscopic insights, the heterostructure is analyzed through electron diffraction methods. Also, the crystallinity comparison through electron diffraction can be authenticated over X‐ray diffraction, as a lateral specimen volume is probed by the electron beam.

**Figure 3:**
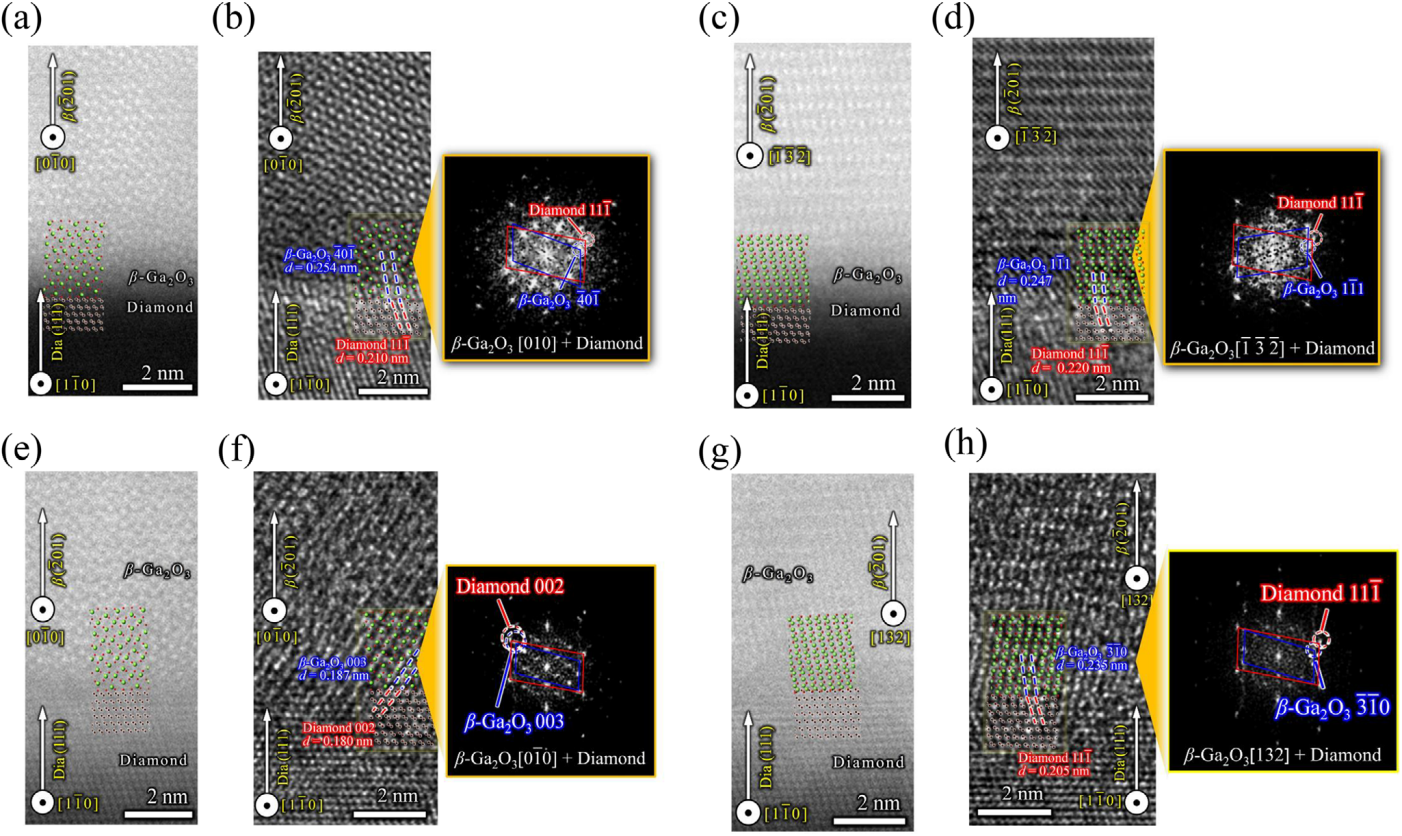
ADF‐STEM images of the a) *β*‐Ga_2_O_3_<010>//diamond [1$\bar{1}$0] c) *β*‐Ga_2_O_3_<132>//diamond [1$\bar{1}$0] epitaxial arrangements, b,d) are the corresponding HR‐TEM images, of the heterostructure grown at 50 W RF power. e–h) are the ADF‐STEM and HR‐TEM images of the same heteroepitaxial arrangements obtained from the heterostructure grown at 35 W RF power. Insets of b,d,f,h) show the epilayer‐substrate merged FFT patterns at the interface of the *β*‐Ga_2_O_3_/diamond (111).

**Figure** **3** shows the high‐resolution electron microscopic images of the 50 and 35 W grown *β*‐Ga_2_O_3_/diamond (111) interface cross‐section, with diamond [1$\bar{1}$0] collinear with the electron beam. The HR‐TEM images disclosed two epitaxial orientations of the *β*‐Ga_2_O_3_ films on the diamond (111) surface with *β*‐Ga_2_O_3_<010>||diamond [1$\bar{1}$0] and *β*‐Ga_2_O_3_<132>||diamond [1$\bar{1}$0]. These distinct arrangements are expected owing to the asymmetric hexagonal array of O atoms on *β*‐Ga_2_O_3_ ($\bar{2}$01) with two side distances bonded to the symmetric hexagonal carbon network. For clarity, the distinct hexagonal arrangements of O‐atoms with respect to diamond [1$\bar{1}$0] will henceforth be referred to as the <010> and <132> domains, with diamond [1$\bar{1}$0] parallel to *β*‐Ga_2_O_3_ <010> and *β*‐Ga_2_O_3_ <132> respectively. Here, the <010> domains will have two equivalent orientations with *β*‐Ga_2_O_3_ [010] / [0$\bar{1}$0] parallel to diamond [1$\bar{1}$0], while the <132> domains will possess four equivalent orientations with *β*‐Ga_2_O_3_ [132] / [$\bar{1}\;\bar{3}\;\bar{2}$] / [$\bar{1}3\bar{2}$] / [1$\bar{3}$2] parallel to diamond [1$\bar{1}$0]. The HR‐TEM images manifest a superior resolution of the epitaxial structure compared to the ADF‐STEM images, as the contrast observed in the ADF‐STEM images depends on the atomic numbers of the elements present.^[^ ^35^ ^]^ Consequently, the structure of the *β*‐Ga_2_O_3_/diamond interface, which exhibits a substantial disparity in atomic numbers (Ga = 31, O = 8, C = 6), was not as discernible as in the TEM image.

The Fast Fourier Transformation (FFT) patterns obtained at the interfaces demonstrated virtual overlap for the epilayer and substrate diffraction spots. Due to the fact that the FFT acquisition area is constrained to a very close proximity of the interface, the pattern is encompassed by a notable background signal. However, the background signal for the 35 W grown heterostructure exhibited relatively low background signal interference, which may be due to a more refined interface at low sputtering powers.

There have been numerous studies on the domain structure of *β*‐Ga_2_O_3_ epitaxial films grown on diamond (100) substrates, where in their recent reports, Nandi et al. and Xu et al. have demonstrated the 30° oriented two‐set domain structure (*β*‐Ga_2_O_3_ [132]||diamond [110] and *β*‐Ga_2_O_3_ [010]||diamond [110]) of the epitaxial film with 12‐fold symmetry.^[^ ^21^, ^22^ ^]^ However, apart from a general perception of the growth domains, the microstructure of the inter‐domain grain boundaries, the presence or absence of crystal defects, and the domain growth mechanism of heteroepitaxial *β*‐Ga_2_O_3_ thin films on diamond (111) substrates remain to be elucidated.

**Figure 4:**
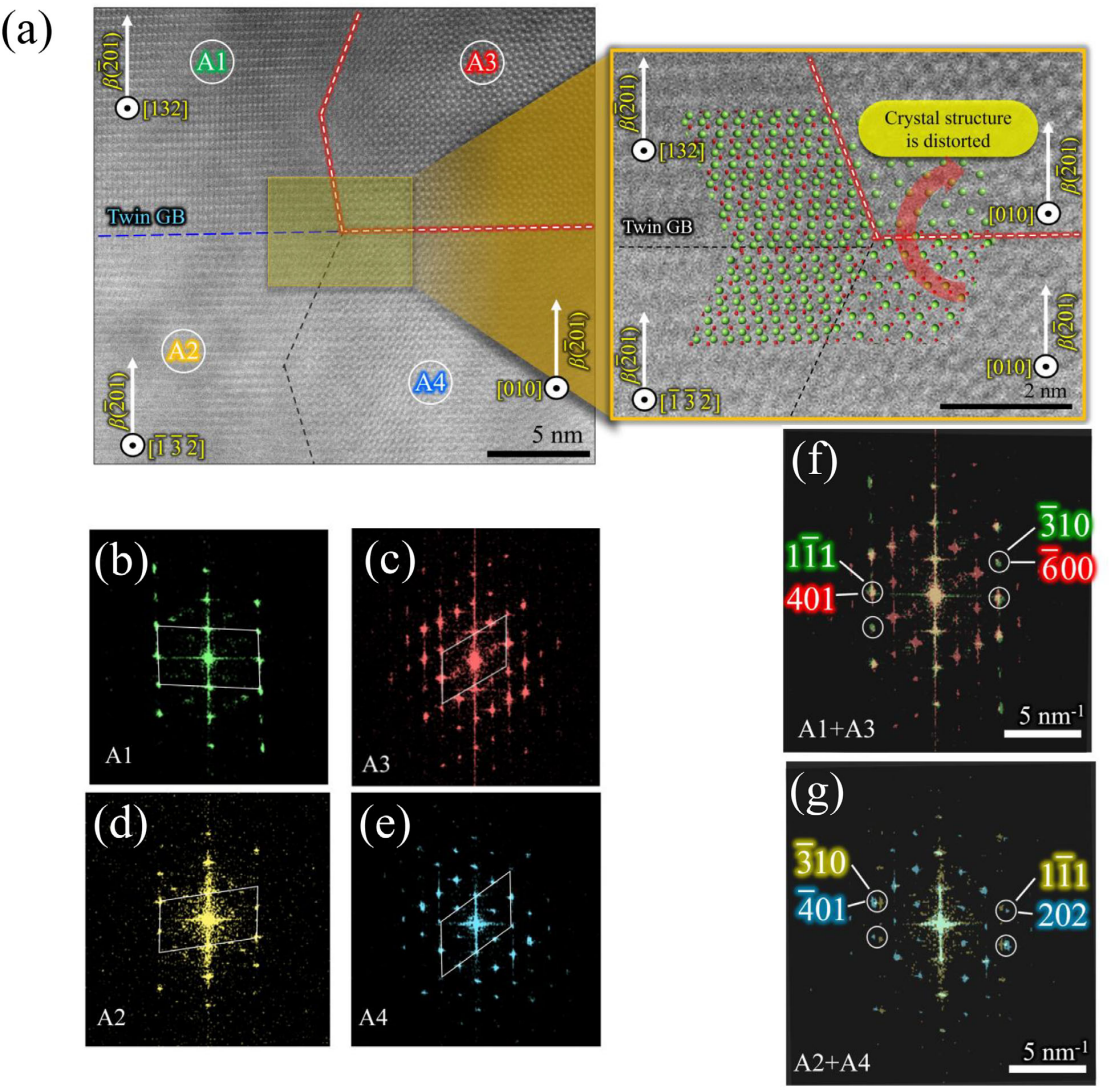
a)Cross‐section ADF‐STEM image of *β*‐Ga_2_O_3_ film at a grain boundary. b–e) are the FFT patterns of areas A1, A2, A3, and A4 marked in (a), respectively. f,g) are the merged FFT patterns of selected areas.

**Figure** **4** shows a cross‐section ADF‐STEM image of the 50 W grown *β*‐Ga_2_O_3_ film at the grain boundary. In this field of view, atomic arrangements of *β*‐Ga_2_O_3_[132], *β*‐Ga_2_O_3_[$\bar{1}\;\bar{3}\;\bar{2}$], and *β*‐Ga_2_O_3_[010] orientations were observed. The electron beam incident direction is diamond [1$\bar{1}$0], similar to Figure 3. The atomic simulation at the heteroepitaxial interface and grain boundaries in this study is performed using the ReciPro Ver.4.896 crystallographic calculation software.^[^ ^36^ ^]^ The presence of a twin grain boundary (GB) is evident between the *β*‐Ga_2_O_3_[132] and *β*‐Ga_2_O_3_[$\bar{1}\;\bar{3}\;\bar{2}$] oriented grains as shown in Figure 4a. However, detailed observation revealed a significant distortion in the crystal structure in the *β*‐Ga_2_O_3_ [010] orientation through the periphery of the expected line of propagation of the twin GB, as illustrated by the red line, which might be due to the GB‐induced strain.^[^ ^37^ ^]^

The FFT patterns corresponding to distinctive areas are displayed in Figure 4b–e. The inference on twin GB formation between *β*‐Ga_2_O_3_[132] and *β*‐Ga_2_O_3_[$\bar{1}\;\bar{3}\;\bar{2}$] oriented grains has been substantiated, as evidenced by the manifestation of inversion symmetry in the respective FFT patterns. The diffraction spots corresponding to (1$\bar{1}$1) and ($\bar{3}$10) have been found to be mirrored about the origin. Also, it has been confirmed that the FFT spots of adjacent crystal grains overlap when they are merged, as shown in Figure 4f,g. In atomic resolution S/TEM observation, the FFT corresponds to the crystal structure itself, and they are generally indicative of the spacing and orientation of the periodic structure.^[^ ^38^ ^]^ Thus, it is found that adjacent grains possess analogous periodic structures. From this analysis, it can be inferred that twin grain boundaries are preferentially formed in <132> domains, while strain‐induced distortion occurs in <010> domains. Consequently, we hypothesize that the crystal growth modes of the *β*‐Ga_2_O_3_<010> domain and the *β*‐Ga_2_O_3_<132> domain may exhibit significant disparities. However, at this point of the discussion, the hypothesis is only supported by local measurements. To confirm the complete authenticity, 4D‐STEM measurements with a large area VDF reconstruction of the *β*‐Ga_2_O_3_ film have been carried out.

**Figure 5:**
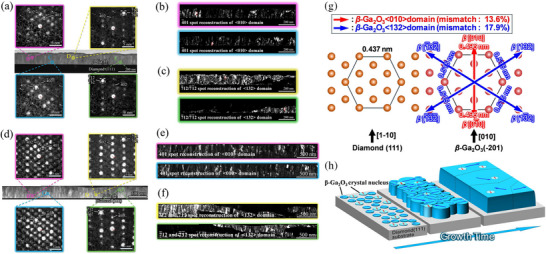
The cross‐sectional ADF‐STEM image of *β*‐Ga_2_O_3_/ diamond (111) interface and local diffraction patterns of *β*‐Ga_2_O_3_ (point B) [010], (point C) [0$\bar{1}$0], (point D) [132] / [$\bar{1}3\bar{2}$], (point E) [$\bar{1}\;\bar{3}\;\bar{2}$] / [1$\bar{3}$2] oriented grains of a) 50 W and (d) 35 W RF power grown heterostructure. The VDF reconstructions of <010> and <132> domains 50 W b,c) and 35 W e,f) grown heterostructure. All component figures share the same color code. g) growth directions of *β*‐Ga_2_O_3_<010> and <132> domains. h) The growth model of heteroepitaxial *β*‐Ga_2_O_3_ films on diamond (111) substrate with growth time.

**Figure** **5**a,d shows the low magnification cross‐section ADF‐STEM image of the *β*‐Ga_2_O_3_/diamond (111) heterostructure and diffraction patterns of differently oriented grains obtained from local points for heterostructures grown with RF powers of 50 and 35 W, respectively. Local diffraction patterns represent *β*‐Ga_2_O_3_ [010], [0$\bar{1}$0], [132] / [$\bar{1}3\bar{2}$], and [$\bar{1}\;\bar{3}\;\bar{2}$] / [1$\bar{3}$2] in‐plane oriented grains. It can be seen that the diffraction spots become distinct and the relative contrast with the background has been increased for the 35 W grown epilayer when compared to that of a 50 W grown *β*‐Ga_2_O_3_ film, authenticating the crystalline improvement as inferred from X‐ray diffraction analysis. The VDF reconstruction of the <010> domain is achieved with $\bar{4}$01 diffraction spots of the *β*‐Ga_2_O_3_ [010], [0$\bar{1}$0] oriented grains as shown in Figure 5b,e for respective growth powers. The resulting VDF image shows a columnar contrast, suggesting a preferential growth in the vertical direction. Similarly, in the VDF image of <132> domains (Figure 5c,e) reconstructed with $\bar{7}$12 / $\bar{7}\;\bar{1}$2 diffraction spots of the *β*‐Ga_2_O_3_ [132] / [$\bar{1}3\bar{2}$], and [$\bar{1}\;\bar{3}\;\bar{2}$] / [1$\bar{3}$2] oriented grains, it can be seen that the grains follow a uniform spatial growth with no preferential growth direction. The area ratios of <132> slightly increased from 72.4% to 78.2% for 35 W grown epilayer, suggesting decreasing the RF power suppresses the <010> domain forming, but does not give drastic difference in the epitaxial growth mode of *β*‐Ga_2_O_3_ thin films on diamond (111) substrates.

These results actively suggest that the *β*‐Ga_2_O_3_ <132> domains follow in‐plane grain development with a 2D growth mode with respect to the growth time and validates the atomic resolution STEM image in Figure 4, which indicated the growth mode difference between <010> and <132> domains. The *β*‐Ga_2_O_3_ <132> domain has four equivalent orientations within a range of ≈60°–180°, facilitating the intersection of adjacent grains. This is presumed to have resulted in a 2D growth mode in which adjacent grains are merged to be aligned in the same direction during the growth process.

This mechanism is consistent with the concept of oriented attachment growth, in which adjacent grains come to align with the same orientation.^[^ ^39^, ^40^ ^]^ Following this mechanism, crystal grains with similar crystallographic directions align to the same orientation during the growth process. Therefore, it's presumed that the ease of forming oriented attachment growth, which means the ease with which each domain's orientation aligns, affects the growth mechanism. In contrast, the *β*‐Ga_2_O_3_ <010> oriented grains have only inversion domains that possess two equivalent orientations, due to which these domains manifest a vertical growth, resulting in a columnar growth mode. The growth model derived from the aforementioned results is shown in Figure 5g,h.

In our recent report based on indirect analysis methods, it was predicted that the epitaxial arrangement is formed by *β*‐Ga_2_O_3_ {502} // diamond {1$\bar{1}$0} (010 domain) and *β*‐Ga_2_O_3_ {532} // diamond {0$\bar{1}$0} (132 domain) with lattice mismatches of ‐1.584% and 2.183%.^[^ ^26^ ^]^ This atom‐to‐atom model is based on *β*‐Ga_2_O_3_ <010> // diamond <1$\bar{2}$1> and *β*‐Ga_2_O_3_ <132> // diamond <1$\bar{2}$1>. However, in the S/TEM studies, it was revealed that the *β*‐Ga_2_O_3_ ($\bar{2}$01) lateral plane is 30° rotated over the diamond (111), bringing *β*‐Ga_2_O_3_ <010> // diamond <1$\bar{1}$0> and *β*‐Ga_2_O_3_ <132> // diamond <1$\bar{1}$0>. So, the epitaxial arrangement is likely to be formed with are *β*‐Ga_2_O_3_ {502} // diamond {1$\bar{2}$1} and *β*‐Ga_2_O_3_ {532} // diamond {1$\bar{2}$1}, for the <010> and <132> domains, respectively. But the lattice mismatches are significant during the primitive domain matching, as shown in Figure 5g. However, in the case of significant mismatch systems, the epitaxial formation can be exemplarily explained through the domain matching epitaxy (DME) model, where the substrate and film lattice units align in their lowest integer multiplied values to find possible overlapping.^[^ ^41^, ^42^ ^]^ Accordingly, the lattice mismatches calculated for *β*‐Ga_2_O_3_ {502} // diamond {1$\bar{2}$1} and *β*‐Ga_2_O_3_ {532} // diamond {1$\bar{2}$1} are −1.05% to 0.64% respectively. According to DME, defects and dislocations are locally concentrated, and the film stress is relaxed by forming specific domain structures.

The *β*‐Ga_2_O_3_ <010> and <132> are denoted in red and blue arrows, respectively. Six orientation microcrystalline nuclei containing *β*‐Ga_2_O_3_<010> and <132> domains are formed during the initial stage of crystal growth. As growth progresses, each domain shows a distinct growth mode, as evidenced by 4D‐STEM measurements. Consequently, the <010> domain forms columnar 3D grains, while the <132> domain forms horizontal 2D grains with the orientation aligned with a specific one, as denoted by the arrows. This results in the formation of *β*‐Ga_2_O_3_ epitaxial film on diamond (111) substrates, in which the 2D grown <132> domains constitute the predominant part, with the presence of some 3D grown <010> domains. The 2D growth mode is generally preferred when the objective is to grow high‐quality thin films of the single‐crystalline like. For the growth of high quality *β*‐Ga_2_O_3_ films on diamond (111) substrates, to this end, it is imperative to inhibit the formation of *β*‐Ga_2_O_3_ <010> domains and to foster the emergence of *β*‐Ga_2_O_3_ <132> domains.

## 3 Conclusion

In summary, this study succeeded in direct observation of the heteroepitaxial interface of *β*‐Ga_2_O_3_/diamond (111) heterostructure fabricated by RFMS for the first time. The HR‐TEM and ADF‐STEM images, at the *β*‐Ga_2_O_3_/diamond (111) interface have confirmed *β*‐Ga_2_O_3_<010>||diamond [1$\bar{1}$0] and *β*‐Ga_2_O_3_<132>||diamond [1$\bar{1}$0] epitaxial arrangements. In the atomic resolution S/TEM observation of grain boundaries in the *β*‐Ga_2_O_3_ crystal, it was perceived that the *β*‐Ga_2_O_3_ <010> domains restrain from forming twin grain boundaries in contrast with *β*‐Ga_2_O_3_ <132> domains, suggesting different growth modes of the concerned domains. In 4D‐STEM measurements and subsequent large area VDF reconstruction, the *β*‐Ga_2_O_3_ <010> domain showed a preferential 3D growth mode characterized by columnar crystal grains, whereas the *β*‐Ga_2_O_3_ <132> domain demonstrated a horizontally 2D growth mode. 2D growth is generally considered beneficial for thin film growth at the single‐crystal level. Therefore, it has been demonstrated that the control of the growth domains is a pivotal factor in determining the caliber of *β*‐Ga_2_O_3_ thin films. This study offers insights into the production of low‐cost and high‐quality *β*‐Ga_2_O_3_/diamond heterostructures by RFMS for active thermal management and advanced bipolar power rectifiers. The authors, in their coming reports, will strive to achieve domain control by optimizing the RFMS growth parameters and substrate off‐angles.

## 4 Experimental Section

### Heterostructure Fabrication

*β*‐Ga_2_O_3_ thin films were grown on diamond (111) substrates (2 × 2 × 0.3 mm, Sumitomo Corp.) by RFMS through sputtering an undoped Ga_2_O_3_ sintered compact target (4N purity). Prior to substrate introduction into the chamber, the diamond substrates were subjected to ultrasonication in acetone, methanol, and DI water for 5, 5, and 10 min, respectively. The cleaned substrates were then dried with N_2_ gas. After that, the substrates were immediately put into a load‐lock chamber of the RFMS apparatus. After ensuring a base pressure of the main chamber of less than 1.0 × 10^−^⁶ Pa, the diamond substrates were transferred into the main chamber. Then, the diamond substrates in the main chamber were heated up to 700 °C. After that, *β*‐Ga_2_O_3_ films were deposited on the diamond substrates using RF powers of 50 and 35 W. The substrate temperature was maintained at 700 °C throughout the entire deposition process, with the substrate holder undergoing uniform rotation. The growth pressure inside the main chamber was constantly maintained at 1.5 × 10^−1^ Pa through the controlled infusion of Ar gas (15 sccm). The deposition times were 72 and 48 h for 50 and 35 W, respectively. Note that the *β*‐Ga_2_O_3_ deposition was carried out without supplying O_2_ gas.

### Structural Evaluation

Indirect crystal structure analysis was carried out using X‐ray diffraction (XRD) *θ*‐2*θ*, 2*θ*, rocking curve, and pole figure projections using an X‐ray diffractometer (SmartLab, Rigaku Corp.). For local‐structural investigation, chemical bonding states of the deposited films were analyzed using X‐ray photoelectron spectroscopy (XPS, KRATOS AXIS NOVA, Shimazu Corp.). The peak fitting in XPS analysis was calibrated against the C1s main peak centered at 285.0 eV. Direct crystal structural analysis of the heterostructures was carried out via S/TEM technique. For the S/TEM observation, the samples were prepared through the lift‐out method by a dual‐beam focused ion beam (FIB, Thermo Fisher Scientific, Scios) and triple‐beam FIB (MI‐4000L, Hitachi High‐Technologies). The prepared specimen was then observed using a TEM (JEM‐ARM300F2, JEOL. Ltd.) and a STEM (Titan Cubed G2, Thermo Fisher Scientific Inc.). In STEM observation, the annular dark field (ADF) collection semi‐angle was adjusted to be 52–200 mrad. S/TEM with aberration correction facilitates observation at atomic resolution and also allows analysis at high spatial resolution.^[^ ^43^ ^]^ Also, the 4D‐STEM technique enabled the mapping of electron diffraction patterns by scanning an electron probe with a convergence angle of ≈0.6 mrad.^[^ ^44^ ^]^ The 4D‐STEM includes 2D real space and 2D reciprocal space information. This technique facilitates enhanced spatial resolution and expedited acquisition of selected area diffraction patterns in comparison to conventional TEM systems while minimizing electron beam damage, allowing the reconstruction of a virtual dark field (VDF) image using any diffraction pattern for a designated specimen area.

## Conflict of Interest

The authors declare no conflict of interest.

## Data Availability

The data that support the findings of this study are available from the corresponding author upon reasonable request.
